# Beneficial mutation-selection dynamics in finite asexual populations: a free boundary approach

**DOI:** 10.1038/s41598-017-17212-5

**Published:** 2017-12-19

**Authors:** Lionel Roques, Jimmy Garnier, Guillaume Martin

**Affiliations:** 1 BioSP, INRA, 84914 Avignon, France; 2 CNRS – Université Savoie Mont-Blanc, LAMA, F-73000 Chambéry, France; 3 CNRS, ISEM, UMR 5554, 34095 Montpellier, France

## Abstract

Using a free boundary approach based on an analogy with ice melting models, we propose a deterministic PDE framework to describe the dynamics of fitness distributions in the presence of beneficial mutations with non-epistatic effects on fitness. Contrarily to most approaches based on deterministic models, our framework does not rely on an infinite population size assumption, and successfully captures the transient as well as the long time dynamics of fitness distributions. In particular, consistently with stochastic individual-based approaches or stochastic PDE approaches, it leads to a constant asymptotic rate of adaptation at large times, that most deterministic approaches failed to describe. We derive analytic formulas for the asymptotic rate of adaptation and the full asymptotic distribution of fitness. These formulas depend explicitly on the population size, and are shown to be accurate for a wide range of population sizes and mutation rates, compared to individual-based simulations. Although we were not able to derive an analytic description for the transient dynamics, numerical computations lead to accurate predictions and are computationally efficient compared to stochastic simulations. These computations show that the fitness distribution converges towards a travelling wave with constant speed, and whose profile can be computed analytically.

## Introduction

Adaptation under mutation selection and drift is a central process of evolutionary biology. With the advent of experimental evolution and cancer studies, much focus on the dynamics of adaptation has shifted from sexuals to asexuals. While the former remains intensely studied and has equally important applications, direct studies of adaptation as it unfolds is often restricted to asexual model species (reviewed in^1^). This new focus has shed light on the complex interplay of drift, selection and mutation in asexuals, especially when multiple lineages co-segregate and compete for ultimate fixation, i.e. with clonal interference (reviewed in^2^).

The simplest microscopic model describing this interplay of drift selection and mutation considers a set of *K* haploid genotypes, in a population of constant census size *N*. Each genotype *i* ∈ [1, *K*] has Malthusian fitness *m*_*i*_, which is the expected rate of change *m*_*i*_ (per unit time) of the log of the genotype’s census size. The population size is maintained constant at size *N* by randomly sampling individuals after reproduction (culling of the population). There are continuous time and discrete time versions of this model (detailed e.g. in^3^): in the continuous time version (Moran Model), reproduction and culling occur asynchronously at exponentially distributed time intervals, in the discrete time model (Wright-Fisher model, used in our simulations), reproduction and culling of the whole population occur synchronously every generation. In both case, mutations arise as a Poisson process with rate *U* per unit time per individual, independently of the genotype. Single mutations produce a mutant offspring which fitness is the sum of the parental fitness and a random deviate, which distribution is independent of the genotype (we assume it has finite moments, as is classically considered). Multiple mutations have additive effect on the offspring. Thus, this classic additive model ignores epistasis for fitness (e.g. discussed in^4–6^). A classic ‘textbook’ result (e.g.^7^) states that the dynamics of genotype frequencies, in the discrete time model (Wright-Fisher), are well approximated by the dynamics of the continuous time version (Moran model), whenever fitnesses do not vary too much among genotypes, at any time. Overall, for both models, the distribution of Malthusian fitness within the population *p*(*t*, ⋅) admits the same limit as *N* → +∞ and in continuous time. This limit is characterized by the following continuous-time nonlocal integro-differential equation (see e.g.^2,8,9^):

1 $${\partial }_{t}p=U(J* p-p)+(m-\bar{m}(t))p,\,t > \mathrm{0,}\,m\in {\mathbb{R}}\,{\rm{with}}\,\bar{m}(t)={\int }_{{\mathbb{R}}}s\,p(t,s)ds,$$

where the sign $$* $$ stands for the convolution product:

2 $$J* p(t,m)={\int }_{{\mathbb{R}}}J(m-s)\,p(t,s)\,ds\mathrm{.}$$

Assuming no epistasis, the distribution of mutation effects on fitness is constant and is described by the mutation kernel *J* (see^4–6^ for other approaches which account for the effects of epistasis). Thus, the integral term $U\,(J* p-p)$ describes the mutation effects. The term $(m-\bar{m}(t))p$ describes selection with $\bar{m}$ giving the mean fitness in the population at time *t*.

Beneficial mutation-selection dynamics in asexual populations are characterized by a constant asymptotic rate of adaptation *v*_∞_ at large times if the effects of mutation on fitness are non-epistatic, i.e., if these effects are additive as we assume here. This behaviour arises, over large times, in numerical simulations of the microscopic model described above (finite *N*). However, the limit dynamical system described by the integro-differential Eq. (1) does not capture this behaviour (as detailed below). The asymptotic rate of adaptation *v*_∞_ depends, among other things, on the mutation rate *U* and the population size *N*. Current theory gives approximate analytic expressions for the expected asymptotic rate of adaptation, depending on these parameters, with different conclusions and approximations for different parameter ranges (e.g.^10–14^). However, they only provide predictions for the long term behaviour of the system, once it reaches a steady dynamical state with a constant rate of adaptation, which typically take several thousand generations of transient “burn in” before they are reached, thus also limiting their application to empirical dynamics. On the other hand, deterministic models of the form (1), based on an infinite population size assumption (e.g.^15,16^) provide insight on transient behaviours (i.e., before drift starts to act^6^) but fail to predict a constant asymptotic rate of adaptation. A deterministic theory that would describe the transient dynamics of the fitness distribution and its convergence towards the correct stationary regime is therefore still missing.

When the mutation kernel *J* contains some beneficial mutations, due to the infinite population size assumption, the approach (1) becomes unrealistic as *t* becomes large^16^: with this approach, the rate of adaptation $v(t)=\bar{m}\text{'}(t)$ increases exponentially fast, and may even blow-up in finite time if the kernel *J*(*s*) decays exponentially or slower as *s* → + ∞. To overcome this problem, Martin and Roques^6^ proposed a phenomenological approach which consisted in connecting the transient dynamics obtained with (1) with the asymptotic rate of adaptation *v*_∞_. In that respect, they computed the time *τ* such that the rate of adaptation given by model (1) reaches some a priori asymptotic rate of adaptation: $\bar{m}\text{'}(\tau )={v}_{\infty }$. Then, they assumed the fitness distribution to behave as a traveling wave with constant profile for *t* ≥ *τ*: *p*(*t*,*m*) = *p*(*τ*,*m* − *v*_∞_(*t* − *τ*)). This approach is accurate, but not entirely satisfying as it provides no information on the asymptotic rate of adaptation *v*_∞_, which has to be estimated numerically using individual-based simulations, or given by other theories.

When mutations with large effects are rare, or mutation rates sufficiently high–see^14^ and chapter II, part 1.2 in^17^, we can use the following diffusion approximation of the mutation effects

3 $$U\,(J* p-p)\simeq D{\partial }_{m}^{2}p-B{\partial }_{m}p,$$

with *D* = *M*_2_ *U*/2, *B* = *M*_1_ *U* and *M*_1_, *M*_2_ the first and second moments of *J*. A main advantage of this approximation is that diffusion operators are generally more tractable than convolutions as we see in the sequel. The dynamics of *p*(*t*, ⋅) can then be approached by the nonlocal PDE:

4 $${\partial }_{t}p=D{\partial }_{m}^{2}p-B{\partial }_{m}p+(m-\bar{m}(t))p,\,t > \mathrm{0,}\,m\in {\mathbb{R}}\,{\rm{with}}\,\bar{m}(t)={\int }_{{\mathbb{R}}}s\,p(t,s)ds\mathrm{.}$$

This model, with *B* = 0 (which can be assumed without loss of generality, by considering the above equation in a frame moving with speed *B* to the right) has been exhaustively investigated by Alfaro and Carles^15^ who derived an analytical characterization of the dynamics of fitness. Their approach showed that, again, the model is unrealistic as the rate of adaptation $v(t)=\bar{m}\text{'}(t)$ ultimately becomes infinite.

As long as beneficial mutations occurs, the deterministic models (1) and (4) fail to describe the asymptotic rate of adaptation. This failure is related to the infinite speed of propagation of the support of *p*(*t*,*m*) which holds for the diffusion operator $D{\partial }_{m}^{2}p$ or the integro-differential operator $U(J* p-p)$ (as long as *J* contains some beneficial mutations) as a consequence of the strong maximum principle (see e.g.^18^ in the diffusion case). Because of this propagation property, positive concentrations of individuals with arbitrarily large fitnesses are created at each time, and tend to increase by selection, pulling the distribution towards larger fitnesses.

Traditionally, this unrealistic feature of many PDE models is compensated by introducing a cut-off^19^, i.e., a threshold value of concentration *p*_*c*_ > 0 below which individuals cannot grow, which means here that they cannot be selected. Tsimring *et al*.^20^ adopted this cut-off approach, replacing the selection term $(m-\bar{m}(t))p$ by $\chi (p-{p}_{c})(m-\bar{m}(t))p$, where *χ*(⋅) is the Heaviside step function. Using this approach, they focused on the large-time dynamics of fitness. They showed in particular the existence of a traveling wave solution with constant speed. The wave speed can then be computed numerically by solving a system of transcendental equations. However, as the speed depends on the threshold *p*_*c*_, it gives no quantitative information on the actual expected asymptotic rate of adaptation and on the transient dynamics in a population with given mutational characteristics.

Alternative approaches such as stochastic integro-differential or diffusion equations have also been considered by several authors^9,21,22^ to take into account the effects of genetic drift arising from the finite size of the population. These equations are of the form (1) or (4), with an additional term $\sqrt{p(t,\,m)}\eta (t,\,m)$ where *η*(*t*, *m*) is Gaussian white noise. Using a stochastic diffusion equation of this form, and some results in^23^, Neher and Hallatschek^14^ established a simple formula for the asymptotic rate of adaptation *v*_∞_, depending on the population size *N*.

The aim of our work is to propose a deterministic framework to follow the transient and large-time dynamics of fitness distributions in finite populations, in the presence of beneficial (and deleterious) mutations. Contrarily to cut-off approaches, this new framework does not rely on the assumption that highly fit mutants do not grow if their concentration is small: here, the best fitness class remains finite at all times. This approach is inspired by the physics of ice melting, where a free boundary separates the liquid and solid phases, which is known as “the Stefan problem”^24^. In our case, the free boundary is a moving interface corresponding to the position of the fittest class at time *t*. Beyond this interface, unlike what happens in the standard diffusion case or with cut-off approaches, the distribution is exactly 0.

### Free boundary approach

In this section, we construct a model which captures some properties of the microscopic models mentioned above, while preserving the PDE formalism of the limit system (4). In particular the support of the distribution is bounded to the right and the rate of adaptation remains finite and depends on the population size *N*. In that respect, we replace the problem (4) by the free boundary problem:

5 $$\{\begin{array}{ccl}{\partial }_{t}p & = & D{\partial }_{m}^{2}p+(m-X(t))p,\,t > \mathrm{0,}\,m\in (-\infty ,s(t))\\ p(t,s(t)) & = & \mathrm{0,}\,t > 0\\ s^{\prime} (t) & = & -\mu \,D\,{\partial }_{m}p(t,s(t)),\,t > 0.\end{array}$$

The free boundary *s*(*t*) separates the region where the distribution is positive with the portion of the fitness space [*s*(*t*), +∞) where the distribution is 0. This free boundary propagates with the speed *s*′(*t*). In the absence of selection (i.e., if (*m* − *X*(*t*))*u* is removed), the Eq. (5) corresponds to the Stefan problem^24^ that we mentioned in the Introduction. Note that this model admits an extra-parameter *μ* compared to (4). This parameter describes how the speed of the interface *s*′(*t*) and the slope of the distribution at this interface are connected. Since *p* is a probability distribution, its mass should equal 1 at all time, leading to the following definition of *X*(*t*):

6 $$X(t)={\int }_{-\infty }^{s(t)}y\,p(t,y)dy-\frac{1}{\mu }\,s^{\prime} (t)=\bar{m}(t)-\frac{1}{\mu }\,s^{\prime} (t),\,t > 0.$$

To our knowledge, there is no generic theory that gives existence and uniqueness of the solution of the Cauchy problem (5)–(6) with initial condition *p*_0_ (but see^25^ for some results on closely related equations, without the nonlocal term *X*(*t*)). We show in Section 2.1 that for each value of the parameter *μ* > 0 the problem (5)–(6) admits a traveling wave solution, whose profile and speed depend on *μ*. We then derive in Section 2.2 an additional relationship between the parameters in (5) and the population size *N*. In particular, we derive a formula for the parameter *μ*–and therefore the asymptotic rate of adaptation–depending on the population size *N*. We compute the corresponding asymptotic rate of adaptation.

### Travelling wave solution

We look for a travelling wave solution with constant speed *v*, i.e., a solution of the form *p*(*t*, *m*) = *ϕ*(*m* − *vt*) and *s*(*t*) = *vt* with *ϕ* > 0 on (−∞, 0) and ${\int }_{-\infty }^{0}\varphi (y)\,dy=1.$ Plugging this solution into (5)–(6) gives

7 $$\{\begin{array}{l}D\,\varphi ^{\prime\prime} (z)+v\varphi ^{\prime} (z)+(z-{\int }_{-\infty }^{0}y\varphi (y)\,dy+\frac{1}{\mu }\,v)\varphi (z)\,=0,\,z\in (-\infty ,0),\\ \varphi \mathrm{(0)}=0\,{\rm{and}}\,\varphi ^{\prime} \mathrm{(0)}=-\frac{v}{\mu \,D}\mathrm{.}\end{array}$$

Then we can compute an explicit solution of this equation using the Airy function Ai which, by definition, solves the ODE Ai″(*z*) − *z*Ai(*z*) = 0, and is bounded in ${\mathbb{R}}$. The solution of (7) is then given by

8 $$\varphi (z)=\frac{v}{\mu \,{D}^{\mathrm{2/3}}\,{\rm{Ai}}\text{'}(-{z}_{0})}{e}^{-\frac{vz}{2D}}{\rm{Ai}}(-{z}_{0}-z/{D}^{\mathrm{1/3}}),$$

where −*z*_0_ ≈ −2.34 is the largest zero of the Airy function (Ai(−*z*_0_) = 0). Additionally, we have $\bar{z}={\int }_{-\infty }^{0}y\,\varphi (y)\,dy=-{D}^{\mathrm{1/3}}{z}_{0}+\frac{v}{\mu }-\frac{{v}^{2}}{4D}\mathrm{.}$ The function $\varphi$ defined by (8) solves the problem (7), it is positive on (−∞, 0) and it is of mass 1 if and only if

9 $$\mu =\frac{v}{{D}^{\mathrm{1/3}}\,{\rm{Ai}}\text{'}(-{z}_{0})}{\int }_{0}^{\infty }{e}^{\frac{vz}{2{D}^{\mathrm{2/3}}}}{\rm{Ai}}(z-{z}_{0})\,dz\mathrm{.}$$

As the above function $v\mapsto \mu$ is strictly increasing, equal to 0 at *v* = 0 and converges to +∞ as *v* → +∞, this shows that for any *μ* > 0, there is a unique *v* > 0 satisfying (9). Altogether, this shows that, for any *μ* > 0, the problem (5)–(6) admits a unique positive travelling wave solution *p*(*t*, *m*) = *ϕ*(*m* − *vt*) satisfying ${\int }_{-\infty }^{0}\varphi (y)\,dy=1.$

### Effect of the population size *N* on the model parameters

**Figure 1:**
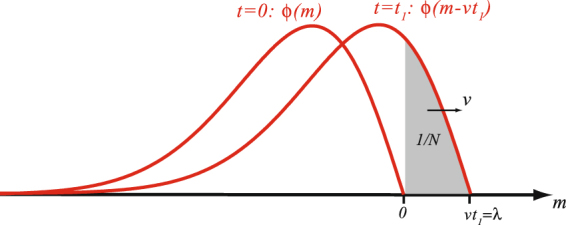
Computation of the time *t*_1_ required to establish a beneficial mutation beyond the initial best fitness class *m* = 0 at time *t* (*t* = 0 here). During the period (*t*, *t* + *t*_1_), the wave travels to the right with speed *v*. The time *t*_1_ is such that the shaded region has mass 1/*N*, i.e., the expected number of individuals with positive fitness is equal to 1.

Assume that the asymptotic rate of adaptation *v*_∞_ has been reached, with a solution of (5)–(6) given by the travelling wave *p*(*t*, *m*) = *ϕ*(*m* − *vt*) with speed *v* = *v*_∞_. In order to derive an additional relationship between *v*, *μ* and the population size *N*, we first compute the expected time *t*_1_ required to establish a beneficial mutation beyond the initial best fitness class at time *t*. Then, we compute the speed as the ratio between the expected increase *λ* in the best fitness class due to this beneficial mutation and the time *t*_1_ (Fig. 1).

For the sake of simplicity, and without loss of generality, we assume that *t* = 0, so that the best fitness class (i.e., the upper bound of the support of the distribution *p*(0, ⋅)) is *m* = 0. At each time *t*, the whole wave *p*(*t*,*m*) is of mass 1, and corresponds to *N* individuals. As the wave moves with speed *v*, the time *t*_1_ is such that the mass between *z* = 0 and *z* = *vt*_1_ is equal to 1/*N*. In other terms:

10 $$N\,{\int }_{0}^{v{t}_{1}}\varphi (m-v{t}_{1})\,dm=N{\int }_{-v{t}_{1}}^{0}\varphi (z)\,dz=1.$$

Approaching *ϕ*(*z*) by *zϕ*′(0) in a neighborhood of 0, we simply get

11 $$N\,{\int }_{-v{t}_{1}}^{0}-\frac{v}{\mu \,D}\,z\,dz=\mathrm{1,}$$

leading to the following expression for *t*_1_:

12 $${t}_{1}=\frac{1}{v}\,\sqrt{\frac{2\mu D}{Nv}}\mathrm{.}$$

Second, we compute the size *λ* of the fitness step implied by the establishment of the beneficial mutation, given that it has occurred. To do so, we use the fact that we are assuming a regime of pervasive clonal interference (many mutations of small effect co-segregate), which is already required for the diffusion approximation used to describe mutation. In this context (detailed in^21^), the backgrounds that produce those mutant lineages destined to invade in the future, tend to be tightly packed at the very rightmost edge of the fitness distribution. In our context, this means that (i) we can assume that the fitness step is due to a beneficial mutation from a background standing at the free boundary, and (ii) the resulting mutants are sufficiently fit and close to one another that their probability of avoiding initial loss due to drift is equal among lineages. Under these two assumptions, the expected fitness increase by these mutants is simply the average fitness effect of beneficial mutations from a parent in the current best fitness class (*m* = 0), so that

13 $$\lambda ={\int }_{0}^{\infty }s\,J(s)ds/({\int }_{0}^{\infty }J(s)ds),$$

(*λ* ≈ (*M*_2_)^1/2^ under the diffusion approximation). We deduce that the asymptotic rate of adaptation *v* = *v*_∞_ should also solve $v=\frac{\lambda }{{t}_{1}}$. Using formula (12), we conclude that:

14 $$v=\frac{2\,D\,\mu }{{\lambda }^{2}\,N}\mathrm{.}$$

Third, we plug the formula (14) into (9), leading to:

15 $${\int }_{0}^{\infty }{e}^{\frac{\mu {D}^{\mathrm{1/3}}}{{\lambda }^{2}N}z}{\rm{Ai}}(z-{z}_{0})\,dz=\frac{{\lambda }^{2}\,N\mathrm{Ai}\text{'}(-{z}_{0})}{2{D}^{\mathrm{2/3}}}\mathrm{.}$$

As the left-hand side in this expression is an increasing function of *μ*, it can easily be solved numerically. Setting $\alpha =\frac{\mu {D}^{1/3}}{{\lambda }^{2}N}$, and approaching the integral in the left-hand side of (15) by exp(*αz*_0_ + *α* ^3^/3), we also get an approximate analytic expression for *μ*: setting $\beta =\frac{{\lambda }^{2}NAi^{\prime} (-{z}_{0})}{2{D}^{2/3}}$, and $\gamma =12\,\mathrm{ln}\,\beta +4\sqrt{{z}_{0}^{3}+9\,{(\mathrm{ln}\beta )}^{2}}$ we get:

16 $$\mu =\frac{{\lambda }^{2}N}{{D}^{\mathrm{1/3}}}[\frac{1}{2}{\gamma }^{\mathrm{1/3}}-2\,{z}_{0}{\gamma }^{-\mathrm{1/3}}]\approx \frac{{\lambda }^{2}N{\gamma }^{\mathrm{1/3}}}{2{D}^{\mathrm{1/3}}},$$

where the last approximation assumes that *β* >> 1, i.e., (*λ*/*U*)^2/3^ *N* >> 1. Using Eq. (14), we get that the corresponding formula for the asymptotic rate of adaptation is:

17 $${v}_{\infty }=v={(\gamma {D}^{2})}^{\mathrm{1/3}}\mathrm{.}$$

This shows that the asymptotic rate of adaptation tends to increase with *N*, like (*lnN*)^1/3^. This is consistent with the findings of Neher and Hallatschek^14^; in the framework of stochastic PDEs, they obtained the following formula:

18 $${v}_{NH}={D}^{\mathrm{2/3}}\,{[24\mathrm{ln}(N{D}^{\mathrm{1/3}})]}^{\mathrm{1/3}}\mathrm{.}$$

As a byproduct of formula (17), we can compute the expected asymptotic variance in fitness *V*_∞_ within a population. Multiplying by *z* the Eq. (7) satisfied by *ϕ* and integrating by parts, we get:

19 $${V}_{\infty }={\int }_{-\infty }^{0}{z}^{2}\varphi (z)\,dz-{\bar{z}}^{2}={v}_{\infty }-\frac{{v}_{\infty }}{\mu }\bar{z}\mathrm{.}$$

Thus, *V*_∞_ can be computed explicitly in terms of the model parameters *λ*, *U*, *N*, *D*, using (17) and the relationship *v*_∞_/*μ* = 2*D*/(*λ* ^2^ *N*) which follows from (14).

## Validation

To check the validity of our approach, we used an individual-based, discrete time Wright-Fisher model of genetic drift, selection and mutation as a benchmark. In this model, each individual *i* = 1, …, *N* has Malthusian fitness *m*_*i*_, with corresponding Darwinian fitness ${e}^{{m}_{i}}$ (discrete time counterpart of the Malthusian fitness). At each generation, *N* individuals are sampled with replacement and according to their Darwinian fitnesses. This is modelled by a multinomial experiment with *N* trials and event probabilities $({e}^{{m}_{1}},\ldots ,{e}^{{m}_{N}})/\sum _{i=\mathrm{1,}\ldots ,N}{e}^{{m}_{i}}$. Then, in this discrete time approach, the mutation step occurs simultaneously in all the individuals. It is simulated by randomly drawing, for each individual, a Poisson number of mutations, with a rate *U*. The effect of a unique mutation on fitness was drawn according to the distribution *J*, multiple mutations having additive effects on the fitness of the offspring. We considered kernels for which the diffusion approximation $U\,(J* p-p)\simeq D{\partial }_{m}^{2}p$ can be made: *J*(*s*) ∝ exp(−|*s*/($\sigma \sqrt{2}$)|^*β*^), with *β* = 2 (Gaussian distribution, as in^14^) and *β* =  = 10 (as in^13^), with small variance 2*σ* ^2^Γ(3/*β*)/Γ(1/*β*) = 10^−4^ (same value as in^14^).

For mutation rates *U* ranging from 10^−4^ to 2 ⋅ 10^−1^, and for population sizes ranging from *N* = 10^2^ to *N* = 10^7^, we simulated 100 replicate trajectories of the individual-based model, for *t*∈(0,1000). We then followed the empirical distribution of fitness in each population. This allowed us to compute the mean fitness ${\bar{m}}_{num}(t)$ within each population, and its average value $< {\bar{m}}_{num}(t) >$ among the 100 replicates.

### Stationary rate of adaptation and distribution of fitness

The stationary rate of adaptation *v*_*num*_ was estimated by computing the slope given by a linear fit of $< {\bar{m}}_{num}(t) >$ for *t*∈(700,1000). The accuracy of the analytical approximation *v*_∞_ in Eq. (17) was checked by computing the ratio *v*_∞_/*v*_*num*_. In the case *β* = 2 (Gaussian mutation kernel), our formula was compared to that in^14^, see Eq. (18). In the case *β* = 10, our formula was compared to that in^13^. More precisely, for beneficial mutation kernels of the form:

20 $$\rho (x)=\frac{1}{\sigma }\frac{{e}^{-{(s/\sigma )}^{\beta }}}{\Gamma \mathrm{(1}+\mathrm{1/}\beta )},$$

with *β* ≫ 1 the following approximation was derived in^13^ (formula (21) in^13^):

21 $${v}_{G}=\frac{2\,{\sigma }^{2}\,\mathrm{ln}(N\sigma \sqrt{\mathrm{ln}(N\sigma )})}{\mathrm{ln}\,{(\frac{\sigma \sqrt{\beta }}{{U}_{b}}\sqrt{\mathrm{ln}(\frac{\sigma \sqrt{\beta }}{{U}_{b}})})}^{2}}{(1+\frac{\mathrm{ln}(\frac{\sigma \sqrt{\beta }}{{U}_{b}})}{4\mathrm{ln}(N\sigma )})}^{-2},$$

where *U*_*b*_ = *U*/2 is the rate of beneficial mutations.

**Figure 2:**
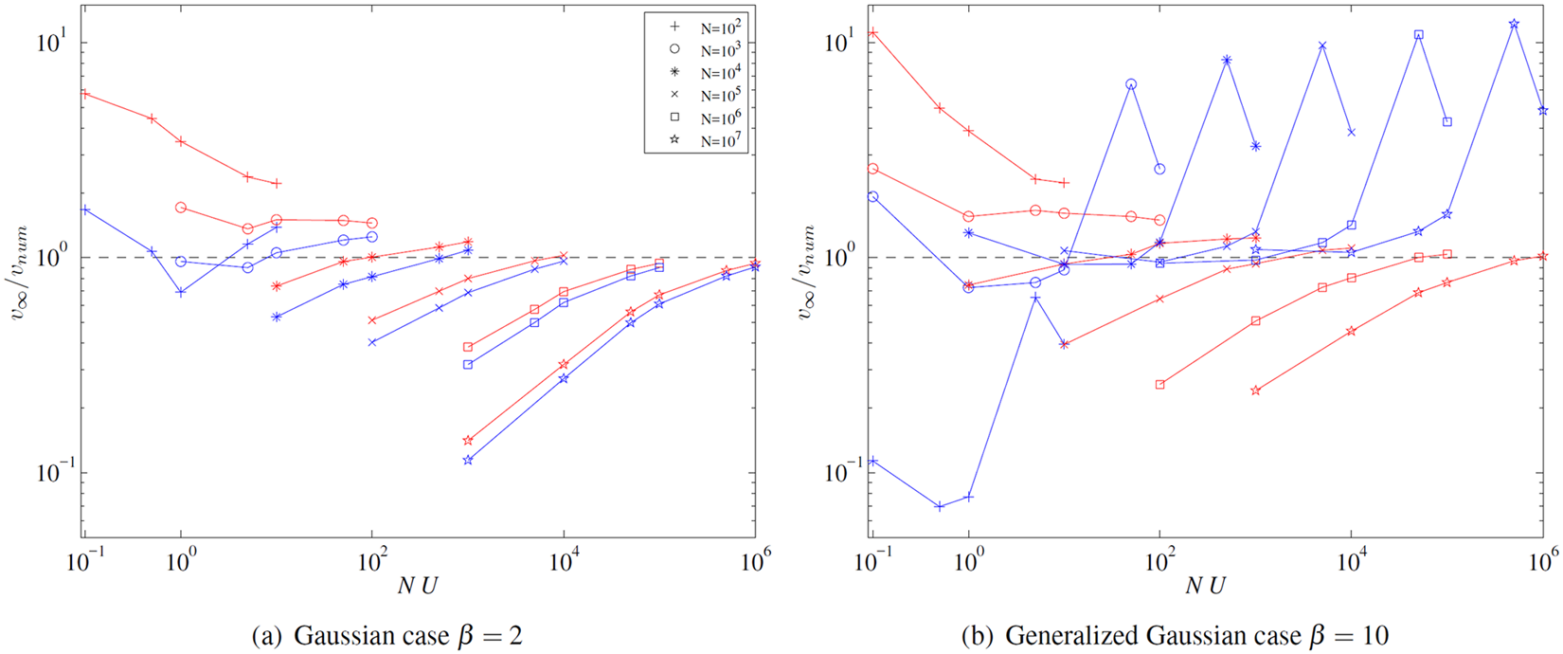
(**a**) (Gaussian case *β* = 2) Red curves: relative error *v*_∞_/*v*_*num*_ between the asymptotic rate of adaptation *v*_∞_ given by our theory (17) and the empirical asymptotic rate of adaptation *v*_*num*_ given by individual-based simulations, depending on the population size *N* and the mutation rate *U*. Blue curves: relative error *v*_*NH*_/*v*_*num*_, where v_*NH*_ is given by (18), corresponding to the theory in^14^ (**b**). (Generalized Gaussian case *β* = 10) Red curves: same legend as in panel a. Blue curves: relative error v_*G*_/*v*_*num*_, where *v*_*G*_ is given by (21), corresponding to the theory of^13^.

The results are presented in Fig. 2(a,b). In the case *β* = 2 (Fig. 2a) we observe that our formula (17) and the formula (18) of Neher and Hallatschek^14^ give accurate results in the sense that *v*_∞_, *v*_*NH*_ and *v*_*num*_ have the same order of magnitude, for all values of *N*, *U*. The asymptotic rate of adaptation *v*_*num*_ is overestimated for small *NU*, while it tends to be underestimated for larger *NU*. Our formula is slightly more accurate than that in^14^ for *NU* > 100, whereas it tends to be less accurate for smaller values of *NU*. For *N* ≥ 10^4^, we observe a clear improvement of the accuracy of formula (17) as *U* increases.

In the case *β* = 10 (Fig. 2b), the accuracy of the formula (17) and its behaviour in terms of *U* and *N* are comparable to the case *β* = 2. Compared to the formula *v*_*G*_ (21), ours tends to be more accurate for large *U* (*U* > 10^−2^) and less accurate for small *U* (*U* < 10^−2^).

The accuracy of the formula (19) for the stationary variance in fitness *V*_∞_ was tested with the same model parameters. The obtained results are comparable in terms of accuracy and of dependence of the accuracy with respect to model parameters, to those described above for *v*_∞_. These results are presented in Supplementary Fig. S1.

**Figure 3:**
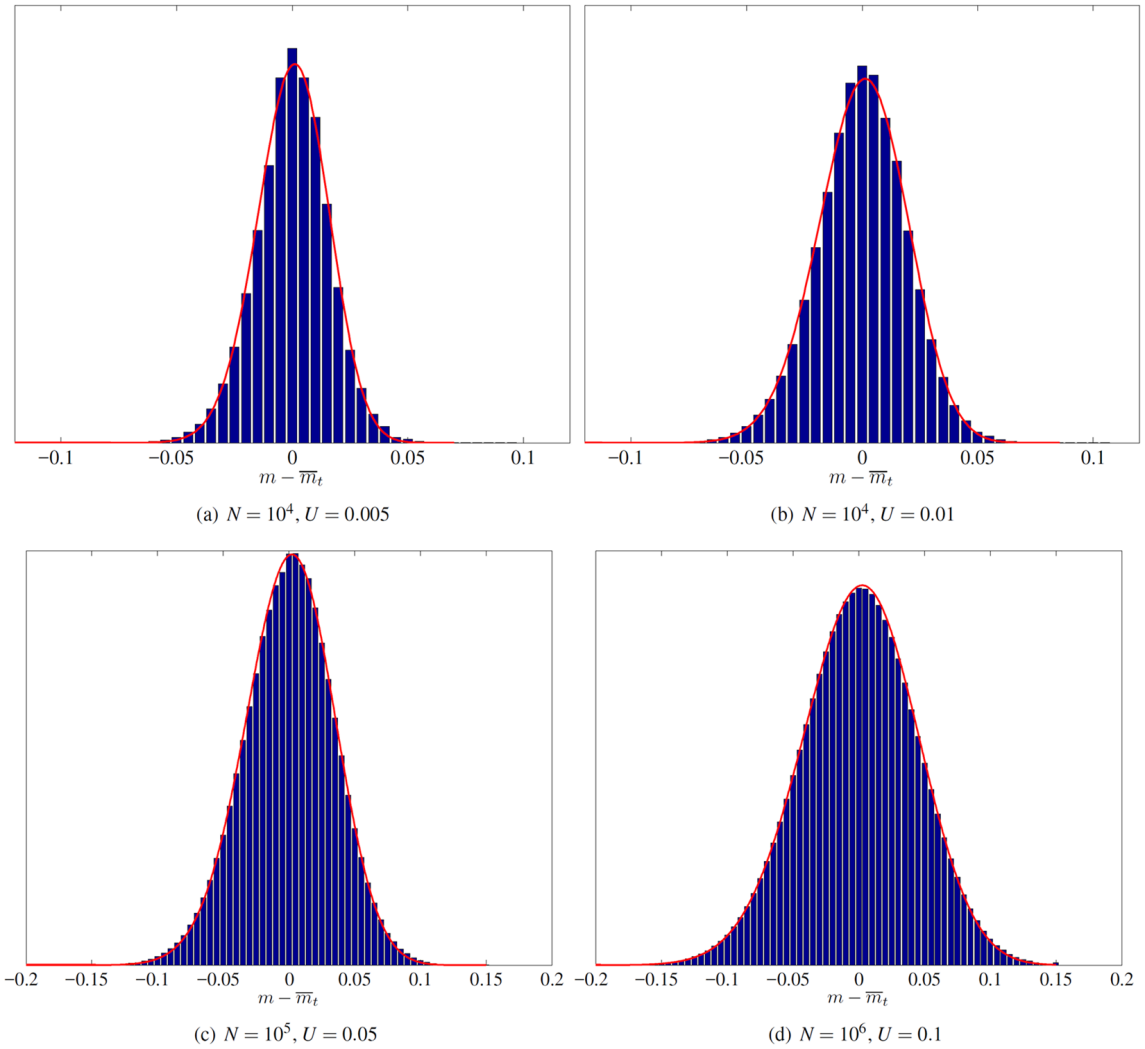
Asymptotic distribution of fitness. Blue histogram: empirical distribution of fitness, centered at 0 and averaged over 100 replicate populations at time *t* = 500, with a Gaussian kernel *J* with variance *σ* ^2^ = 10^−4^. Red line: analytic expression (8), with parameters *μ* and *v* given by (16)–(17).

Regarding the full asymptotic distribution of fitness, we compared the analytic description (8), with parameters *μ* and *v* given by (16)–(17), with empirical distributions obtained by individual-based simulations at time *t* = 500, averaged over the 100 replicates. This comparison was carried out with four sets of parameters *N*, *U*, corresponding to values *v*_∞_/*v*_*num*_ close to 1 in Fig. 2. The results presented in Fig. 3 show that, in the four cases considered here, our theory provides a very precise description of the expected asymptotic distribution.

### Trajectories of adaptation

One of the main points of this work is to derive full trajectories of adaptation, connecting the initial state (here, a Dirac distribution at *m* = 0) with the stationary regime of adaptation given by the travelling wave described in Section 2.1. Using the formula (16) for setting the value of *μ* in the free boundary dynamical system (5), we were able to get a numerical solution *p*(*t*,*m*), using a standard finite difference method (Matlab^®^ source code is provided in Supplementary File S3). We compared the trajectories of the mean fitness $\bar{m}(t)$ and of the expected variance in fitness $V(t)={\int }_{{\mathbb{R}}}{s}^{2}\,p(t,s)\,ds-{\bar{m}}^{2}(t)$ with the empirical mean $< {\bar{m}}_{num}(t) >$ of ${\bar{m}}_{num}(t)$ and of the variance in fitness given by individual-based simulations with a Gaussian mutation kernel (*σ* ^2^ = 10^−4^). All of the simulations were carried-out during the time-interval *t*∈(0,1000). Solving the Eq. (5) needed about 0.5 second, whereas it took about 18 hours to compute the empirical mean of $\bar{m}(t)$, based on 100 replicate populations with *N* = 10^6^ individuals, and 6 days with *N* = 10^7^ (on an Intel(R) Xeon(R) CPU E5-2637 v3 @ 3.50 GHz).

**Figure 4:**
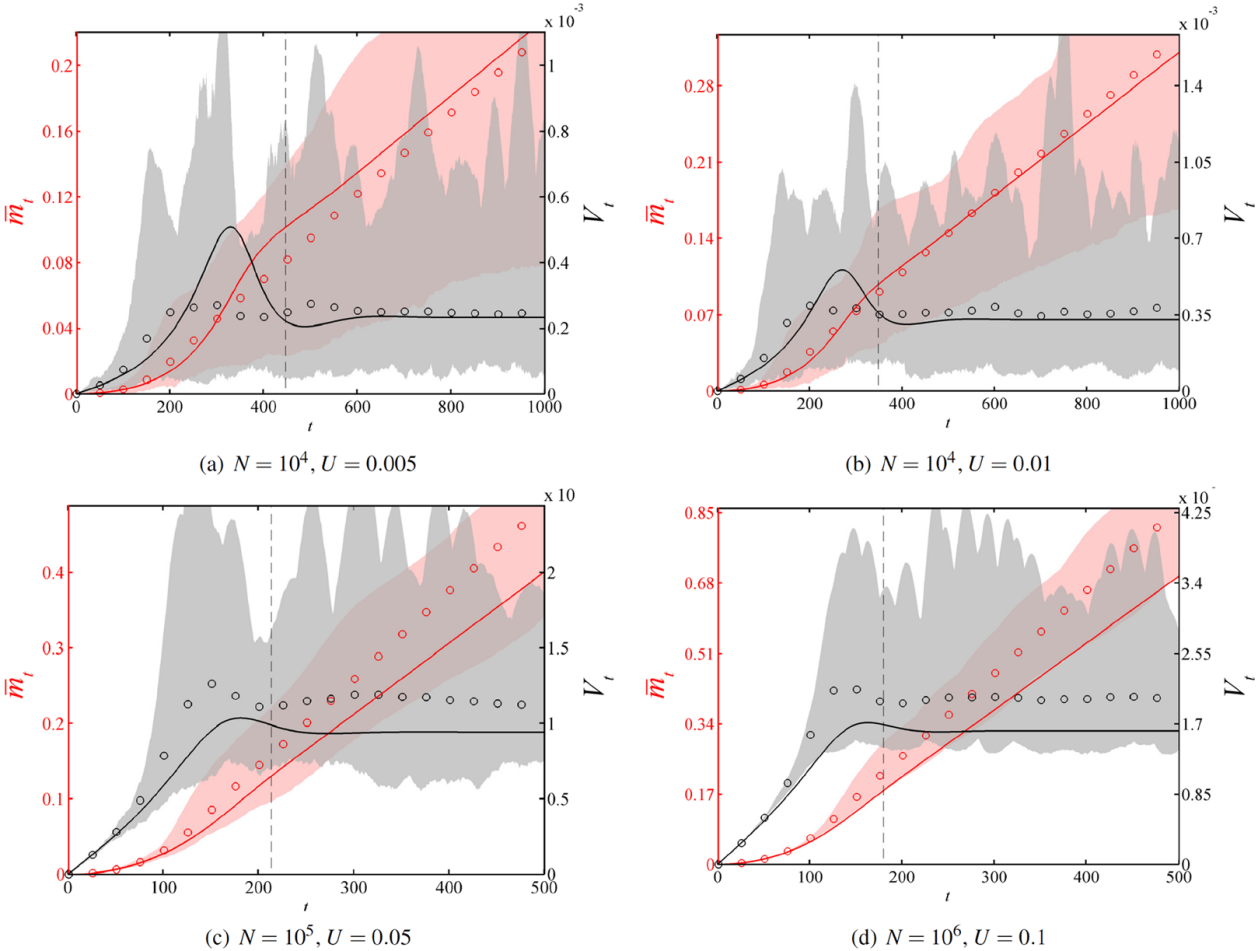
Mean fitness $\bar{m}(t)$ and variance trajectories *V*(*t*). Plain lines: values of $\overline{m}(t)$ (in red) and $V(t)={\int }_{{\mathbb{R}}}{s}^{2}\,p(t,s)\,ds-{\bar{m}}^{2}(t)$ (in black) given by numerically solving (5)–(6). Circles: empirical mean fitness $< {\bar{m}}_{num}(t) >$ and variance given by individual-based simulations averaged over 100 populations, with a Gaussian kernel *J* with variance *σ* ^2^ = 10^−4^. Shaded regions: 99% confidence intervals for the mean fitness (in red) and the variance (in gray). The vertical dashed line corresponds to the characteristic time *t*_*q*_ given by formula (22), such that a proportion *q* = 0.95 of the asymptotic rate of adaptation is reached.

We depicted four trajectories of adaptation in Fig. 4, corresponding to values *v*_∞_/*v*_*num*_ close to 1 in Fig. 2. Contrarily to other deterministic approaches including beneficial mutations^15,16^, the rate of adaptation $v(t)=\bar{m}\text{'}(t)$ converges towards a constant value. Although it was expected, this does not directly follow from the existence of a travelling wave with constant speed *v*_∞_, as this travelling wave could have been unstable. Additionally, the approach captures the emergence of a transient maximum in variance, which was not the case with the approach proposed in.^6^

As a byproduct of formula (17), we can compute the characteristic time *t*_*q*_ that it takes to reach a proportion *q* of the speed *v*_∞_. For the classical diffusion problem (without free boundary), starting from a clonal population at *m* = 0, we get $\bar{m}(t)=D\,{t}^{2}$ ^15^. Thus, $\bar{m}\text{'}({t}_{q})=q\,{v}_{\infty }$ implies

22 $${t}_{q}=q\,{v}_{\infty }\mathrm{/(2}\,D\mathrm{).}$$

The accuracy of this formula is also illustrated in Fig. 4.

The numerical solution of Eqs (5)–(6) also provides the expected dynamics of the full distribution of fitness. Convergence towards a traveling wave is illustrated in Supplementary Fig. S2. The accuracy of the fitness distribution predicted by the free boundary approach (5)–(6) with parameter *μ* given by (16) is illustrated in Supplementary Video 4; it is compared to an empirical distribution obtained by individual-based simulation, with *N* = 10^4^ and *U* = 0.01. The distribution predicted by the standard integro-differential model (1) (same Gaussian kernel as in the individual-based model) is illustrated in Supplementary Video 5 for comparison.

## Discussion

Using an analogy with an ice melting problem, we proposed a deterministic PDE framework which captures the entire dynamics of fitness distribution in the presence of beneficial mutations with non-epistatic effect. Our approach allows for an approximate treatment of genetic drift, while retaining the useful properties of previous deterministic approximations of these dynamics: a full description of the fitness distribution, at all times, even in the presence of multiple co-segregating alleles. On the other hand, as in existing stochastic models, the introduction of the free boundary, driven by stochastic effects at the “nose” of the fitness distribution, leads to a constant asymptotic rate of adaptation, which order is consistent with that given by stochastic individual-based simulations, even with small population sizes (e.g., *N* = 10^3^).

This framework led to new analytic formulas for the asymptotic rate of adaptation *v*_∞_ and for the asymptotic distribution of fitness *ϕ*. The formula (17) for *v*_∞_ depends on the population size v like (*ln*(*N*))^1/3^, which is consistent with previous results obtained in the framework of stochastic PDEs^14,23^. The accuracy of this formula is comparable to that in^13^ and^14^ for a wide range of population sizes and mutation rates. Yet, the description of genetic drift, in the present approach, is much more simplified than in these previous stochastic PDE models. That both methods yield similar accuracy for long term asymptotic behaviour, suggests that the heuristic used here is sufficient to deal with genetic drift in the free boundary framework.

Regarding the trajectories of adaptation, our theory predicts that the fitness distribution converges towards a travelling wave with speed *v*_∞_, whose profile corresponds to the asymptotic distribution of fitness *ϕ* mentioned above. The numerical simulations of Section 3.2 indicate that, as soon as the formula for *v*_∞_ is accurate, our approach also successfully captures the transient dynamics of the expected mean fitness and the expected variance in fitness. Although we were not able to derive an analytic description for the transient dynamics, the numerical computation of the solution of our free boundary model was very fast compared to stochastic simulation models. It should therefore be more efficient for computationally intensive studies, in particular for parameter estimation (e.g., estimation of the mutation rate) and model validation, based on experimental data.

Free boundary problems have already been developed to analyze the dynamics of other *N*-particle systems^26–28^, such as *N*-Branching Random Walks and *N*-Branching Brownian Motions introduced by Brunet and Derrida^19,29^. In these systems, all of the particles reproduce at the same rate, independently of their positions, and the leftmost particle is removed at each birth event. These assumptions naturally lead to free boundary problems in the limit *N* → +∞, with a free boundary situated on the left (opposite side of the direction of propagation). Our approach is fundamentally different due to the nature of the *N*-particle system that we consider. In the Wright-Fisher model of selection and genetic drift (i) the “drift” step consists in a uniform sampling of individuals (or particles) to form the next generation, leading to a limit system which is not a free boundary problem; (ii) the selection step implies that individuals with larger fitness (i.e., larger value on the *m*-axis) have more offspring on average, by definition of fitness. This leads to a limit system which does not capture the qualitative dynamics of the finite particle system, even for *N* large (finite vs infinite speed of propagation), although the limit system does give a good approximation of the dynamics over a limited period of time, which increases as *N* gets larger, see^6^. Therefore, the free boundary model that we constructed here is more a “mesoscopic” or intermediate scale model than an infinite *N* limit of a *N*-particle system. The model captures some properties of the finite particle system (upper bound to the support of the distribution, finite rate of adaptation, dependence of this rate with respect to *N*) while preserving the PDE formalism of the limit system. As *N* → +∞, its properties become analogous to the initial PDE limit system (*v*_∞_ → +∞ and *s*′(*t*) → +∞), emphasizing its mescoscopic nature.

The approach developed here assumes no epistasis, leading to unbounded fitness trajectories. Saturating fitness trajectories (sublinear increase) are typically observed in long term evolution experiments (e.g. reviewed in^30^). These patterns are consistent with pervasive “diminishing returns” epistasis^31^, i.e., combinations of beneficial mutations are less advantageous than their summed effects would predict. Such trajectories can be generated if one assumes that there is a fitness optimum, *m* ^*^^6^. In such case, by definition, beneficial mutations cannot go beyond the optimal fitness, thus the diffusion term $D{\partial }_{m}^{2}p$ in Eq. (5) must be replaced by a “context-dependent” mutation term, i.e., a mutation term which depends on the current fitness *m*. Examples of such operators include integral terms of the form $U\,{\int }_{{\mathbb{R}}}{J}_{y}(m-y)p(t,y)\,dy-U\,p(t,m)$, where *J*_*y*_ is the mutation kernel, given the fitness *y* of the parent (the kernel is then supported in (−∞,*m* ^*^ − *y*]). However, to the best of our knowledge, there is no theory for dealing with such nonlocal operators in a free boundary framework.

Finally, we believe that the formal approach which was developed here may be generalized to other frameworks, for the derivation of deterministic approximations of a finite population systems. For instance, to compute correction terms for the speed of propagation of traveling wave solutions of Fisher-KPP type equations, as in^19,32^.

## Electronic supplementary material

Supplementary Figures S1 S2

Supplementary Video S4

Supplementary Video S5

Supplementary File S3
