# Supplementary material for: Beneficial mutation-selection dynamics in finite asexual populations: a free boundary approach
Source: Sci Rep. 2017 Dec 19;7:17838. doi: 10.1038/s41598-017-17212-5 (PMC5736637; doi:10.1038/s41598-017-17212-5)
Supplement: Supplementary file 1 — Supplementary Figures S1 S2 [file 41598_2017_17212_MOESM1_ESM.pdf]

Beneficial mutation-selection dynamics in finite asexual populations: a free boundary approach

Lionel Roques[1,*], Jimmy Garnier[2], and Guillaume Martin[3]

[1]BioSP, INRA, 84914, Avignon, France
[2]CNRS – Université Savoie Mont-Blanc, LAMA, F-73000 Chambéry, France
[3]CNRS, ISEM, UMR 5554, 34095 Montpellier, France
[*]lionel.roques@inra.fr

Supplementary Figure S1: Asymptotic variance in fitness

With the same computations as those of Section 3 (main text), we tested the accuracy of formula (16) (main text) for the stationary variance in fitness V_∞. The empirical stationary variance in fitness V_{num} is computed as the average of the variance in fitness at time $t = 1000$, the average being computed among 100 replicate populations.

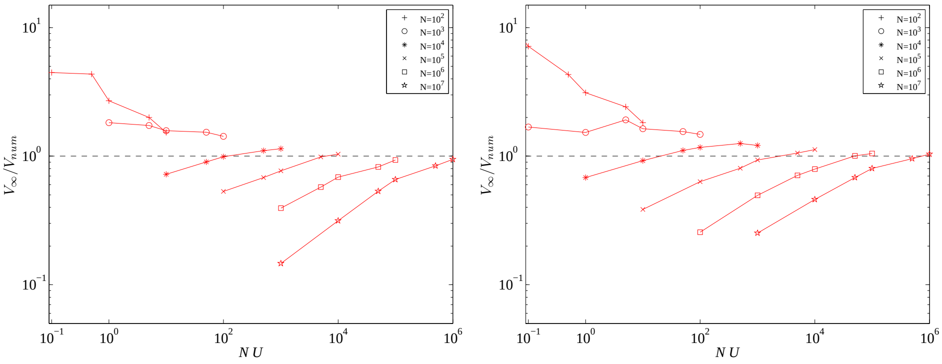

(a) Gaussian case $\beta = 2$ (b) Generalized Gaussian case $\beta = 10$

Figure 1: Relative error V_∞/V_{num} between the stationary variance in fitness V_∞ given by our theory (16) (main text) and the empirical value computed at time $t = 1000$, and averaged over 100 replicate populations.

Supplementary Figure S2: Convergence towards travelling waves

The numerical computation of the solution $p(t, m)$ of equations (4)-(5) (main text) shows that it converges towards a travelling wave with constant speed and constant profile. This is illustrated in the figure below.

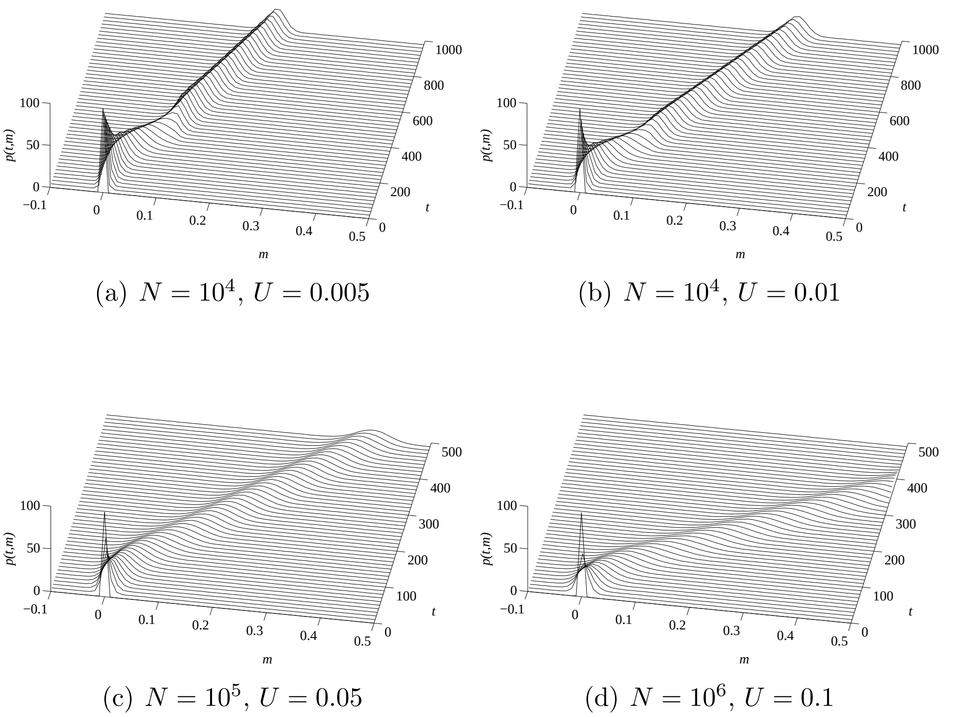

(a) $N = 10^4$, $U = 0.005$

(b) $N = 10^4$, $U = 0.01$

(c) $N = 10^5$, $U = 0.05$

(d) $N = 10^6$, $U = 0.1$

Figure 2: Expected dynamics of the full distribution of fitness given by (4)-(5) (main text).
